# Systemic Toxicity Reported for CDK8/19 Inhibitors CCT251921 and MSC2530818 Is Not Due to Target Inhibition

**DOI:** 10.3390/cells8111413

**Published:** 2019-11-09

**Authors:** Mengqian Chen, Jing Li, Jiaxin Liang, Zanshé S. Thompson, Katie Kathrein, Eugenia V. Broude, Igor B. Roninson

**Affiliations:** 1 Department of Drug Discovery and Biomedical Sciences, College of Pharmacy, University of South Carolina, Columbia, SC 29208, USA; chenm@cop.sc.edu (M.C.); jl13@email.sc.edu (J.L.); jiaxin_liang@dfci.harvard.edu (J.L.); broude@cop.sc.edu (E.V.B.); 2 Department of Biomedical Engineering, College of Engineering and Computing, University of South Carolina, Columbia, SC 29208, USA; zanshe@email.sc.edu; 3 Department of Biology, University of South Carolina, Columbia, SC 29208, USA; klk@sc.edu

**Keywords:** CDK8, CDK19, kinome profiling, zebrafish toxicity assays, STAT1 serine phosphorylation

## Abstract

CDK8/19 kinases, which mediate transcriptional reprogramming, have become an active target for cancer drug discovery. Several small-molecule CDK8/19 inhibitors showed in vivo efficacy and two have entered clinical trials, with no significant toxicities reported. However, Clarke et al. (eLife 2016; 5; e20722) found severe systemic toxicity associated with two potent CDK8/19 inhibitors, Cmpd3 (CCT251921) and Cmpd4 (MSC2530818), and suggested that their toxicity was due to on-target effects. Here, we compared five CDK8/19 inhibitors: Cmpd3, Cmpd4, Senexin B, 16-didehydro-cortistatin A (dCA) and 15w, in different assays. Only Cmpd4 showed striking toxicity in developing zebrafish. In cell-based assays for CDK8 and CDK19 inhibition, Cmpd3, Cmpd4, dCA and 15w showed similar low-nanomolar potency and efficacy against CDK8 and CDK19, while Senexin B was less potent. Only dCA produced sustained inhibition of CDK8/19-dependent gene expression. While toxicity of different compounds did not correlate with their effects on CDK8 and CDK19, kinome profiling identified several off-target kinases for both Cmpd3 and Cmpd4, which could be responsible for their toxicity. Off-target activities could have been achieved in the study of Clarke et al. due to high in vivo doses of Cmpd3 and Cmpd4, chosen for the ability to inhibit STAT1 S727 phosphorylation in tumor xenografts. We show here that STAT1 S727 phosphorylation is induced by various cytokines and stress stimuli in CDK8/19-independent manner, indicating that it is not a reliable pharmacodynamic marker of CDK8/19 activity. These results illustrate the need for careful off-target analysis and dose selection in the development of CDK8/19 inhibitors.

## 1. Introduction

The Mediator kinases, CDK8 and its closely related paralog CDK19, have become active targets for drug development, primarily for oncological applications [1]. Unlike better-known members of the cyclin-dependent kinase (CDK) family, such as CDK1, CDK2 and CDK4/6, CDK8/19 do not mediate cell cycle progression but are only involved in transcription [2,3]. Furthermore, in contrast to the best-known transcriptional CDKs, such as CDK7 and CDK9, CDK8/19 activity is not required for overall transcription, and CDK8 or CDK19 depletion do not inhibit proliferation of most cell types [4]. Instead, CDK8/19 regulate transcription in a selective gene-specific context, primarily enhancing the induction of silent genes as they become activated by various transcription factors [5]. This unique function defines CDK8/19 as mediators of transcriptional reprogramming, a process that plays a key role in the plasticity of tumor cells [3,5], allowing these cells to colonize heterologous environment (metastasis) and survive adverse conditions (drug resistance). Indeed, CDK8 knockdown or CDK8/19 inhibition in colon cancer selectively suppressed the growth of metastatic but not primary tumors [6], suppressed invasive growth by counteracting epithelial-mesenchymal transition [7], interfered with damage-induced secretion of proteins enhancing tumor growth and drug resistance [8], primarily through an effect on NFκB-induced transcription [5], and suppressed the development of resistance to estrogen deprivation in estrogen-receptor positive breast cancers [9]. The ability of CDK8/19 inhibitors to suppress metastatic growth and to prevent the development of therapy resistance suggests a potentially transformative potential for combinations of these inhibitors with various cancer therapeutic regimens. 

A number of small-molecule CDK8/19 inhibitors have been developed [1]; such inhibitors showed in vivo therapeutic effects in leukemia [10,11], lung [8], breast [9], colon [6] and prostate cancers [12]. Notably, these effects were associated with no apparent systemic toxicity [6,9,10,11]. The first clinical trials of Mediator kinase inhibitors have been initiated in estrogen-receptor positive breast cancers (ClinicalTrials.gov Identifier: NCT03065010) and acute myeloid leukemia (AML) (ClinicalTrials.gov Identifier: NCT04021368).

Despite the encouraging results with several CDK8/19 inhibitors, a different picture emerged from a report by Clarke et al. [13]. In this and preceding studies [14,15,16], authors developed two chemically distinct series of small molecule inhibitors with reportedly high selectivity and potency for CDK8/19, starting from cell-based assays for β-catenin inhibition. The two most advanced compounds in both series, Cmpd3 (CCT251921) and Cmpd4 (MSC2530818), were then tested in a battery of in vitro and in vivo assays. Doses of these compounds used for in vivo studies were selected to provide sustained inhibition of the phosphorylation of transcription factor STAT1 at S727 in the treated tumors, which was assumed by the authors to be a pharmacodynamic (PD) marker of CDK8/19 inhibition. Although these compounds showed efficacy in colon cancer and leukemia mouse xenograft models, the authors also noted detectable weight loss in mice. When Cmpd3 and Cmpd4 were tested in rats and dogs, they yielded multiple and striking toxicities, including lethality, which were overlapping but not identical between the two compounds. The authors concluded that the observed toxicities were on-target effects of CDK8/19 inhibition and even suggested that CDK8/19 inhibition should be screened against in the development of future CDK inhibitors [13].

The results of Clarke et al. stand at odds to the studies by other groups that did not observe toxicity in mouse tumor models and brought CDK8/19 inhibitors to the clinical stage following testing in other species. Furthermore, some of the specific phenotypes reported by Clarke et al. [13] disagree with the results reported for other CDK8/19 inhibitors. In particular, transcriptomic analysis of Colo-205 xenografts treated with Cmpd3 or a related compound CCT251545 revealed effects on gene expression that were entirely different from those induced in the same xenograft model by a compound with no reported toxicity [11]. In another case, Clarke et al. reported that Cmpd3 and Cmpd4 adversely affected bone development in vitro (Cmpd3) and induced significant bone pathology (Cmpd3 and Cmpd4). In contrast, another group [17] found the opposite effect with different CDK8/19 inhibitors, which acted as anticatabolic agents to impede excessive osteoclastogenesis and promoted cancellous bone regeneration, suggesting therapeutic potential of such inhibitors to restrict osteolysis and enhance bone regeneration. Furthermore, 15k, a compound now known to be a CDK8/19 inhibitor [17,18], was orally administered to ovariectomized rats over 6 weeks and induced significant improvements in areal bone mineral density, without any reported toxicities such as those observed by Clarke et al. in this species [19]. 

It is unknown whether the toxicity of Cmpd3 and Cmpd4 of Clarke et al. could be due to off-target effects, to a selective effect on either CDK8 or CDK19, or to the high potency or stability of CDK8/19 inhibition at the doses used in that study [13]. In the present study, we have compared Cmpd3 and Cmpd4 with three other CDK8/19 inhibitors in regard to in vivo toxicity, strength and duration of target inhibition in vivo, CDK8 and CDK19 selectivity and off-target activities. We have also investigated whether STAT1 S727 phosphorylation is a suitable PD marker for CDK8/19 activity. Our results indicate that CDK8/19 inhibition is very unlikely to be the cause of toxicity induced by high doses of Cmpd3 and Cmpd4 and reveal major off-target effects of both compounds that could have been responsible for their toxicity. We also show that great caution should be applied in using STAT1 S727 phosphorylation as a PD marker in the development of new CDK8/19 inhibitors. 

## 2. Materials and Methods

### 2.1. Cell Culture, Reagents and Compounds

CDK8/CDK19 single knockout and double knockout derivatives of human embryonic kidney (HEK) 293 cells (ATCC CRL-1573) were generated as previously described [18]. All cells were cultured in DMEM (high-glucose) media supplemented with 10% fetal bovine serum (FBS) and Penicillin-Streptomycin-Glutamine (1×) at 37 °C with 5% CO_2_ and routinely confirmed to be free of mycoplasma. Microscopic examination was carried out by Olympus CKX41 Inverted Phase Contrast Microscope and the cell images were captured and processed with CellSens standard software. 

Human recombinant TNFα (Z00404-50) was obtained from GenScript (Piscataway, NJ, USA). Senexin B and 15w were provided by Senex Biotechnology (Columbia, SC, USA). Didehydro-cortistatin A (dCA) was a gift from Phil S. Baran (Scripps Research Institute, La Jolla, CA, USA). Cmpd3 (CCT251921) and Cmpd4 (MSC2530818) were a gift from Merck KGaA, Darmstadt, Germany. Recombinant human epidermal growth factor (EGF) and transforming growth factor beta (TGFβ) proteins were obtained from R&D Systems, Minneapolis, MN, USA. Other reagents were obtained from MilliporeSigma, St. Louis, MO, USA. 

### 2.2. Zebrafish Toxicity Assays 

The toxicity screen using dechorionated zebrafish embryos (*Danio rerio*, AB strain) was performed by Arkana Laboratories, Little Rock, AR, as a service. The toxicity of different CDK8/19 inhibitors was evaluated in sterile 96-well plates with U-shaped bottoms. 12 wells (i.e., biological replicates) per test chemical and the vehicle control (0.1% DMSO) were used and the experiment was performed in triplicate. Treatment was performed via addition of 20 µL of 10× concentrations of each tested compound in 1% DMSO-containing egg water to each well containing one 24 hpf (hours post fertilization) mechanically-dechorionated embryo in 180 µL volume of egg water (i.e., 0.1% final DMSO concentration). The fish embryos cultured at 28.5 °C were examined at 24, 48, 72 and 96 h post treatment (hpt) and scored as “healthy”, “abnormal” or “dead”. Phenotypes observed and assessed as abnormal included: cardiac edema (intermediate and severe stages), overall edema, enlarged head, malformation of the eyes, curved body axis, shortened body axis and malformation of the tail. Senexin B was tested at 4 µM. dCA, Cmpd3, Cmpd4 and 15w were tested at 1 μM. Positive control 3,4-dichloroaniline (3,4-DCA) was tested at 8 mg/L. 

The toxicity studies in non-dechorionated embryos of zebrafish TU strain were approved by the Institutional Animal Care and Use Committee of the University of South Carolina. The studies were conducted in sterile 24-well plates with one 24 hpf embryo in each well in a 1 mL volume of egg water. For each plate, 6 wells were treated with vehicle control and the other 18 wells (i.e., biological replicates) were treated with different CDK8/19 inhibitors at different concentrations. Fish embryos were cultured at 28.5 °C and monitored daily and the number of healthy embryos was counted daily using the same criteria as in the previous paragraph. All the data from the plates with at least five healthy vehicle-treated embryos on day 5 were compiled together for final data analysis.

### 2.3. RNA Extraction, Reverse Transcription and Quantitative Reverse Transcription-PCR (QPCR)

To evaluate IC50 values for different CDK8/19 inhibitors, 293 cells (wild-type or with the knockout of CDK8, CDK19 or both CDK8 and CDK19) were seeded in 12-well plates at 3 × 10^5^ cells in 1 mL regular culture media per well, 24 h before treatment. Cells were first pre-treated with Senexin B (at 1 μM, 300 nM, 100 nM, 30 nM, 10 nM) or CA/Cmpd3/Cmpd4/15w (at 1 μM, 100 nM, 10 nM, 3 nM, 0.3 nM) or solvent control DMSO (0.1%) for 1 h and then treated with or without 10 ng/mL TNFα for 2 h. In wash-off studies, cells were first pre-treated with Senexin B (1 μM) or CA/Cmpd3/Cmpd4/15w (30 nM) or solvent control DMSO (0.1%) for 3 h before removal of the drug-containing media. Cells were then washed with PBS twice and cultured in fresh regular culture media without any drugs for different periods of time. Total RNA was extracted using RNAeasy Mini Kit (Qiagen, Germantown, MD, USA) and 1 µg of total RNA was used to generate cDNA using iScript cDNA synthesis kit (Bio-Rad Laboratories, Hercules, CA, USA). Gene expression was quantified using iTaq Universal SYBR green super mix using CFX384 Real-Time System (Bio-Rad). The primers used for real-time PCR are listed in Table S1. 

### 2.4. Kinome Profiling, Kd Determination and Off-Target Activities Measurement 

Senexin B, Cmpd3 and Cmpd4 were tested at 2 μM in a high-throughput binding assay (KINOMEscan^TM^, DiscoverX, Fremont, CA, USA) against a panel of 468 kinases. The screening platform employs an active site-directed competition binding assay to quantitatively measure interactions between test compounds and targeting kinases. KINOMEscan™ assays do not require ATP and thereby report true thermodynamic interaction affinities that do not depend on the ATP concentration. To determine dissociation constants (Kds) of compounds on different targets, an 11-point 3-fold serial dilution of each test compound was tested in independent duplicate assays. 

Effects of different compounds on GSK3β kinase activity were evaluated using ADP-Glo^TM^ Kinase Assay kit and GSK3β Kinase Enzyme System (Promega, Madison, WI, USA). Kinase reactions were carried out at room temperature for 60 min. Each reaction contained 1 ng GSK3β enzyme, 25 μM ATP, 0.2 μg/μL GSK3 substrate and test compounds at different concentrations. Equal volume of ADP-Glo reagent was then added to kinase reaction and incubated at room temperature for 40 min, followed by the addition of Kinase Detection Reagent and incubation at room temperature for another 20 min. Bioluminescence signals were measured in 384-well plates using ChemiDoc Touch™ (Bio-Rad) and ImageLab software (Bio-Rad, Version 5.2.1 build 11). Effects of inhibitors were presented as relative kinase activity, calculated from vehicle control signals divided by drug-treated reaction signals. 

To evaluate PIKFYVE kinase inhibition, HEK293-WT and HEK293-dKO cells were treated with CDK8/19 inhibitors at different drug concentrations for 3 h. Phase-contrast microscopy was then used to detect the formation of intracellular large vesicular structures, associated with PIKFYVE inhibition.

### 2.5. Western Blotting 

Cells were cultured in 60-mm dishes and treated under different conditions before being lysed in RIPA lysis buffer with protease/phosphatase inhibitor cocktail. The protein concentration of extracts was determined using the DC protein assay (Bio-Rad Laboratories). Protein samples (50 μg) were resolved on 4–12% Express-Plus PAGE gels in Tris-MOPS (SDS) running buffer (GenScript, Piscataway, NJ, USA), transferred to PVDF membranes and incubated at 4 °C overnight with primary antibodies STAT1 (sc-592, Santa Cruz Biotechnology, Dallas, TX, USA) and pSTAT1-Ser727 (#8826, Cell Signaling Technology, Danvers, MA, USA) followed by anti-rabbit (NA934, GE Healthcare, Chicago, IL, USA) secondary antibodies. Bands were visualized with Western Lighting Plus ECL detection reagent (Perkin Elmer, Waltham, MA, USA) using ChemiDoc Touch™ (Bio-Rad). Images were analyzed using ImageLab (Bio-Rad) and ImageJ (version 1.52p) software for protein signal quantification. 

### 2.6. Statistical Analysis 

To evaluate statistical significance of differences in toxicities of compounds tested in AB strain zebrafish embryos, a two-way repeated measures ANOVA, followed by Dunnett’s multiple comparison test, was performed to analyze results collected at different time points with GraphPad Prism 7.0 (GraphPad Software, San Diego, CA, USA). To assess results from toxicity assays in TU strain zebrafish embryos, risk ratio (RR) and its confidence intervals (CI) for different compounds were calculated using R (version 3.6.1), an open source programming language for statistical computing, with the ‘riskratio’ function in package ‘fmsb’ (version 0.6.3), to determine if inhibitor exposure affects the risk of unhealthy embryo formation. The IC50 values for different inhibitors in inhibiting CDK8/19-dependent gene expression were calculated using a three-parameter least square (ordinary) fitting method with GraphPad Prism 7.0 (GraphPad Software, San Diego, CA, USA). The Kruskal–Wallis non-parametric ANOVA was used to compare gene expression levels under no-inhibitor conditions between wild-type cells and knockout derivatives (8KO, 19KO and dKO) with GraphPad Prism 7.0. One-way ANOVA (assuming Gaussian distribution), followed by Dunnett’s multiple comparison test, was used for comparing normalized GSK3β kinase activities between vehicle control and different inhibitors using GraphPad Prism 7.0.

## 3. Results

### 3.1. CDK8/19 Inhibitors Vary in Their In Vivo Toxicity in Zebrafish Assays

**Figure 1:**
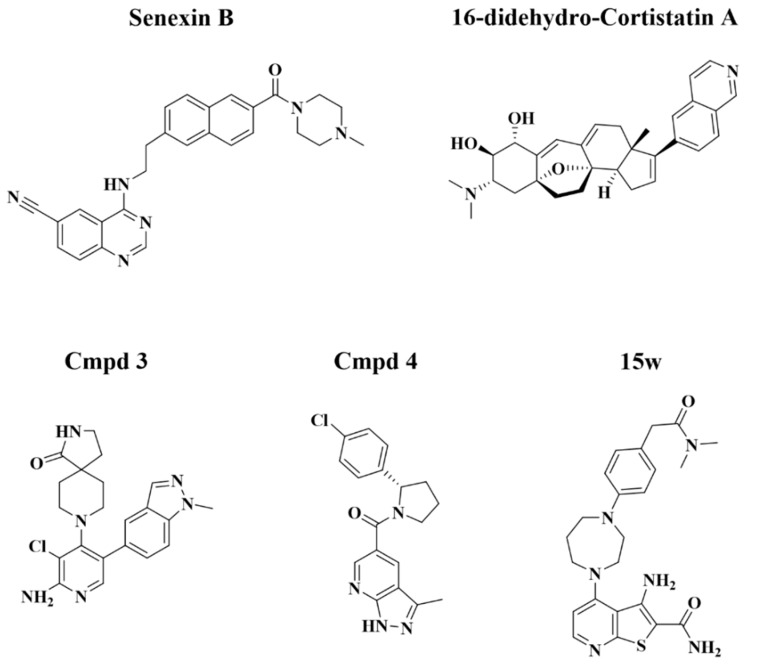
Chemical structures of CDK8/19 inhibitors.

We have assembled a panel of CDK8/19 inhibitors with different chemical structures that were reported to be highly selective for CDK8/19 (Figure 1), including Senexin B [9], the first selective CDK8/19 inhibitor to enter clinical trials (ClinicalTrials.gov Identifier: NCT03065010); Cmpd3 (CCT251921) and Cmpd4 (MSC2530818) of Clarke et al. [13]; 16-didehydro-cortistatin A (dCA), an equipotent version of cortistatin A [20], which was identified by kinome profiling as an inhibitor of CDK8, CDK19, ROCK1 and ROCK2 [21]; and 15w, a thienopyridine recently identified by kinome profiling as a selective CDK8/19 inhibitor, with RIOK2 kinase as its principal off-target [17]. 

**Figure 2:**
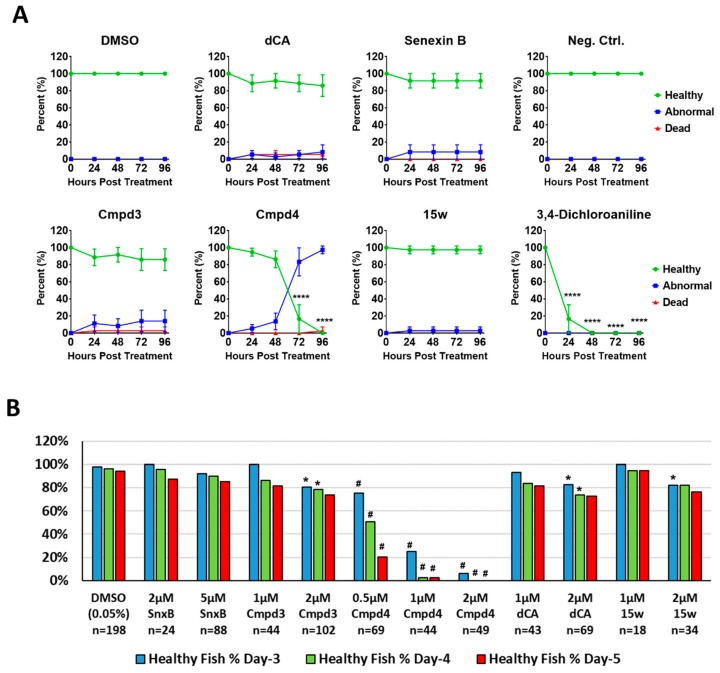
Evaluation of compound toxicity in developing zebrafish. (**A**) Evaluation of compound toxicity using 24 hpf mechanically-dechorionated zebrafish embryos (AB strain). Test compound concentration: vehicle control (0.1% DMSO, *n* = 12); Senexin B (4 µM, *n* = 12); dCA (1 µM, *n* = 12); Cmpd3 (1 µM, *n* = 12); Cmpd4 (1 µM, *n* = 12); 15w (1 µM, *n* = 12); negative control (egg water, *n* = 6); 3,4-dichloroaniline (8 mg/L, *n* = 6) (positive toxicity control). The experiment was performed in triplicate with biological replicates. Fish embryos were examined at 24, 48, 72 and 96 h post compound addition and scored as “healthy”, “abnormal” or “dead”. Statistical significance of difference in percentage of healthy embryos was evaluated by RM two-way ANOVA, followed by Dunnett’s multiple comparison test (****: *p* < 0.0001). (**B**) Evaluation of compound toxicity using 24 hpf non-dechorionated zebrafish embryos (TU strain). Data from multiple technical replicate experiments are pooled together to calculate the overall percentage of healthy normal embryos in the presence of different compounds. Risk ratios (RR) of different treatments compared with vehicle-treated group (DMSO) were calculated to compare risks of unhealthy development under exposure to different inhibitors. Asterisk (*) indicates RR > 5 and pound sign (#) indicates RR > 10. See Table S2 for statistical analysis.

To compare in vivo toxicity of these compounds in the same assay, we used developing zebrafish embryos to perform a toxicological assay that can be conducted rapidly and in small volumes (minimizing the required amounts of each compound), in a sensitive vertebrate organism with excellent tissue permeability [22]. Figure 2A compares the toxicity of different CDK8/19 inhibitors in mechanically dechorionated AB zebrafish embryos, which were examined at 24, 48, 72 and 96 h post treatment (hpt) and scored as “healthy”, “abnormal” or “dead”. Phenotypes observed and assessed as abnormal included: cardiac edema (intermediate and severe stages), overall edema, enlarged head, malformation of the eyes, curved body axis, shortened body axis and malformation of the tail (see examples in Figure S1). In this study, Senexin B was tested at 4 μM, while the more potent compounds dCA, Cmpd3, Cmpd4 and 15w were tested at 1 μM. Positive toxicity control 3,4-dichloroaniline was used at 8 mg/L. Among CDK8/19 inhibitors, Cmpd4 displayed severe toxicity in dechorionated AB zebrafish larvae starting after 72 hpt. Differences between the effects of the other compounds and the negative control did not reach statistical significance in this assay.

Thе same analysis was conducted on a larger scale using TU strain zebrafish embryos without dechorionation. Figure 2B shows the fractions of healthy larvae 3, 4 and 5 days after the addition of different compounds at different concentrations (no noticeable phenotypes were detected after the first two days). Statistical evaluation of the differences between the control and inhibitor-treated zebrafish is presented in Table S2. This analysis confirmed very strong toxicity of Cmpd4 at all the tested concentrations (0.5 μM, 1 μM and 2 μM), followed by Cmpd3 (2 μM) and dCA (2 μM). Hence, CDK8/19 inhibitors with reportedly high selectivity showed wide differences in their in vivo toxicity, with Cmpd4 displaying uniquely high toxicity.

### 3.2. Toxicity of CDK8/19 Inhibitors Does Not Correlate with the Potency or Stability of CDK8 and CDK19 Inhibition in Cell-Based Assays

**Figure 3:**
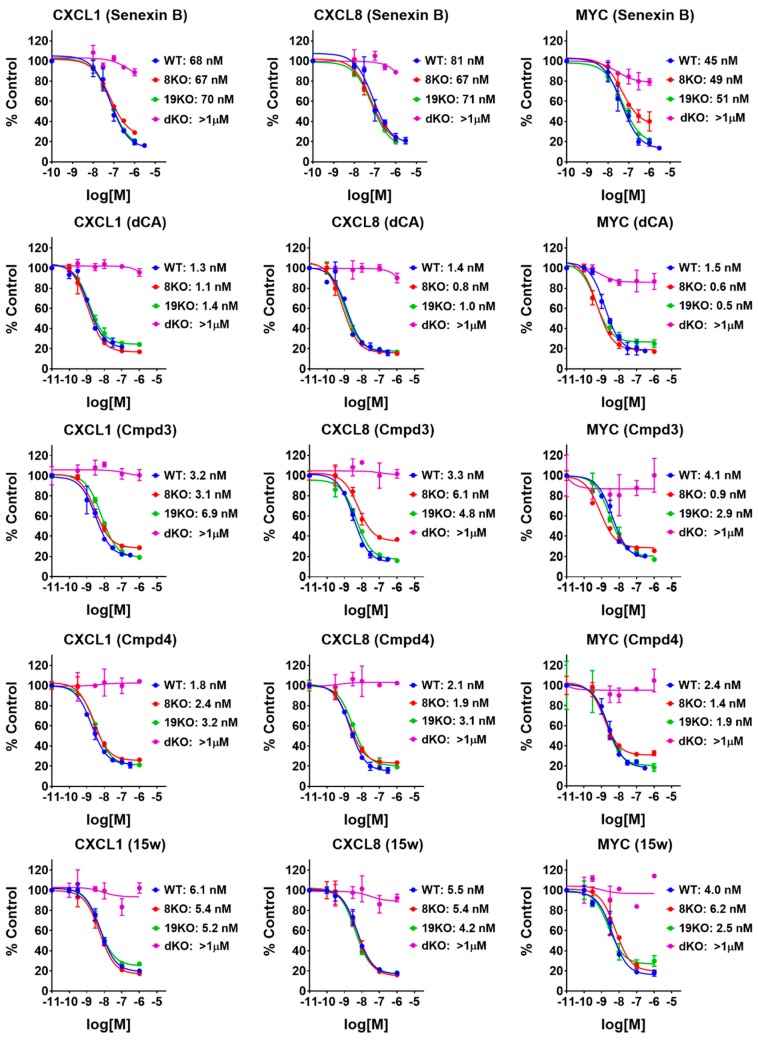
Cell-based assays for CDK8 and CDK19 inhibition by different compounds. HEK293 parental (WT) and knockout (CDK8 single-knockout (8KO), CDK19 single-knockout (19KO) and CDK8/19 double-knockout (dKO)) cells were pre-treated with CDK8/19 inhibitors at five different concentrations for 1 h and then treated with 10 ng/mL TNFα for 2 h (in the presence of the inhibitors) before RNA extraction and QPCR quantification for mRNA expression of CDK8/19-dependent genes. Percentage of inhibition was calculated by normalization to the expression levels in vehicle (0.1% DMSO) control samples. IC50 values are shown on the right of the plots. Data points are presented as mean ± SEM (*n* = 2).

We have used a panel of 293-derived cell lines with CRISPR knockout of CDK8 (8KO), CDK19 (19KO) or both CDK8 and CDK19 (double knockout, dKO) [18], as well as wild type (WT) cells to measure the effects of CDK8, CDK19 and different CDK8/19 inhibitors on gene expression. Cells were pre-treated with different concentrations of CDK8/19 inhibitors for 1 h and then 10 ng/mL TNFα, an inducer of transcription factor NFκB, which is potentiated by CDK8/19 [5], was added for 2 h before RNA extraction. QPCR was carried out to quantify CDK8/19-regulated expression of NFκB-inducible genes CXCL1 and CXCL8 and the basal expression of MYC. In the absence of inhibitors, all three genes showed similar expression in the WT, 8KO and 19KO cells treated with TNFα but were strongly downregulated in dKO cells (Figure S2), indicating that CDK8 and CDK19 are both efficient in regulating these genes. As shown in Figure 3, none of the five CDK8/19 inhibitors affected the expression of the three genes in dKO cells (IC50 >> 1 μM), indicating that their effect on NFκB was CDK8/19 specific. All the inhibitors, however, had very similar effects on the WT, 8KO and 19KO cells (Figure 3), indicating lack of discrimination between CDK8 and CDK19. Four of the inhibitors had similar single-nanomolar activity in this assay, whereas Senexin B was an order of magnitude less active. Hence, the toxicity observed in the zebrafish assays was not associated with a stronger CDK8/19 inhibition or with selective inhibition of one of the two isoforms.

**Figure 4:**
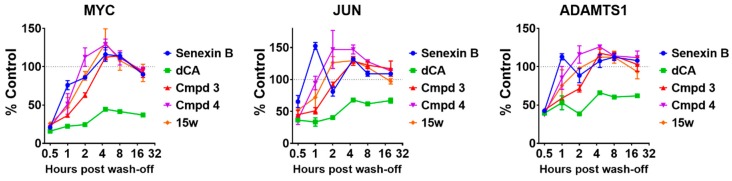
Effects of different CDK8/19 inhibitors on stability of the inhibition of CDK8/19-dependent gene expression in the wash-off study. 293 cells were pre-treated with Senexin B (1 μM) or CA/Cmpd3/Cmpd4/15w (30 nM) or solvent control DMSO (0.1%) for 3 h before removal of the drug-containing media and then incubated with drug-free media for indicated period of time. Percentage of inhibition was calculated by normalization to the expression levels in control samples and presented as mean ± SEM (*n* = 3).

We then asked if such toxicity could be associated with the stability of CDK8/19 inhibition. For this analysis, 293 cells were treated with vehicle (0.1% DMSO), 1 μM Senexin B, 30 nM dCA, 30 nM Cmpd3, 30 nM Cmpd4 or 30 nM 15w for 3 h (these were approximately the lowest concentrations required to achieve maximal inhibition of mRNA levels of MYC, JUN and ADAMTS1 genes that are dependent on CDK8/19 for their inducer-independent expression in 293 cells [5]). Then the drug-containing media were removed, cells were washed twice with PBS and cultured in fresh drug-free media for different periods of time before being lysed for RNA extraction and QPCR analysis of mRNA expression of MYC, JUN and ADAMTS1. Figure 4 shows the time course of the recovery of gene expression after the wash-off of different inhibitors. The results indicate that CDK8/19 inhibition was by far the most stable after treatment with dCA relative to all the other inhibitors. Hence, prolonged inhibition was not a cause of in vivo toxicity. 

### 3.3. Off-Target Kinase Inhibition by Toxic CDK8/19 Inhibitors

**Figure 5:**
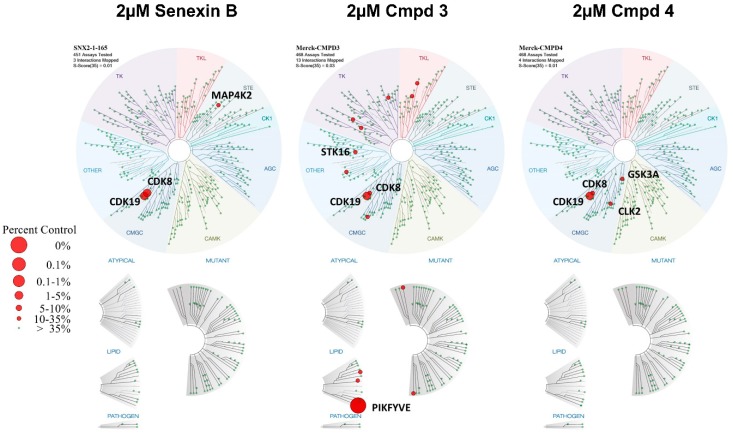
Kinome profiling. Effects of 2 μM Senexin B, Cmpd3 and Cmpd4 on the activity of 468 kinases, measured by an active site-directed competition binding assay, in KINOMEscan profiling (DiscoverX). Kinases showing binding inhibition (< 35% percent of control) are marked with red circles. All the values are presented in Table S3.

To identify off-target activities of Cmpd3 and Cmpd4, we have carried out KINOMEscan profiling (DiscoverX/Eurofins), using an assay that measures the ability of a compound to inhibit the binding of an ATP analog to 468 kinases [23]. This assay has been previously used with many CDK8/19 inhibitors, including cortistatin A and its equipotent analog dCA [20,21]; 15w [17]. Cmpd3, Cmpd4 and Senexin B, which were used at similar doses in mouse in vivo studies [6,9,13,15], were assayed in parallel at 2 μM concentrations. The percent activity of each kinase in the presence of the compounds relative to the control is presented in Table S3. The results of the profiling are displayed in Figure 5 in the form of an evolutionary dendrogram of the kinome, where the size of the red dots represents the magnitude of kinase inhibition. The results of this qualitative screen were confirmed and extended to quantitative measurements by determining Kd binding values for the kinase targets identified by this profiling using KdELECT assay with an 11-point 3-fold serial dilution of each test compound. The results of this analysis are presented in Table 1 and Figure S3.

**Figure 6:**
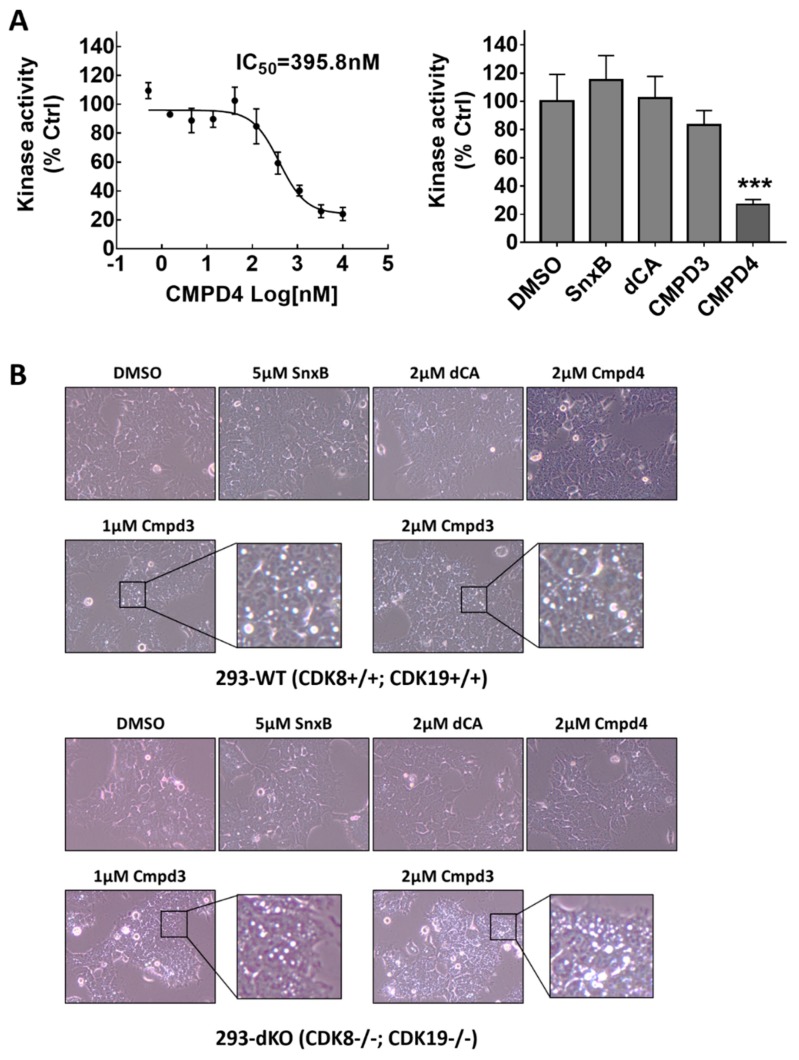
Off-target activity verification. (**A**) Inhibitory effects of CDK8/19-inhibiting compounds at different concentrations on GSK3β kinase activity determined by the ADP-Glo™ Kinase Assay (see Section 2.4). Statistical significance of differences between inhibitor-treated and control conditions is marked by asterisks (***: *p* < 0.001). (**B**) Phase-contrast microscopic images of HEK293-WT and HEK293-dKO cells treated with CDK8/19-inhibiting compounds at indicated drug concentrations for 3 h. Treatment with Cmpd3 causes the formation of intracellular large vesicular structures, as evidence of PIKFYVE kinase inhibition.

The results of this analysis show, surprisingly, that the Kd values for CDK8 and CDK19 binding by Cmpd3 and Cmpd4 (but not Senexin B) are at least an order of magnitude higher than the IC50 values determined in a cell-based assay (Figure 3). The screening results also identified several off-target kinases for both Cmpd3 and Cmpd4, with PIKFYVE being the strongest off-target for Cmpd3 and GSK3B for Cmpd4. Тhe corresponding effects on these off-target kinases were tested in kinase-specific assays. Figure 6A shows the inhibitory effects of different concentrations of Cmpd4 (left) and of single high concentrations of other CDK8/19 kinase inhibitors (right) on GSK3β kinase activity, as determined using the ADP-Glo™ Kinase Assay (Promega). The results show that Cmpd4 inhibits GSK3β with IC50 close to 400 nM while the other CDK8/19 inhibitors do not significantly inhibit GSK3β.

Inhibition of PIKFYVE, a FYVE finger-containing phosphoinositide kinase and the strongest off-target of Cmpd3, was shown to induce the formation of swollen intracellular vesicles [24]. Figure 6B shows phase-contrast microscopic images of WT and dKO 293 cells treated for 3 h with vehicle control (0.1% DMSO), Senexin B, dCA, Cmpd3 or Cmpd4. Treatment with Cmpd3 but not with the other CDK8/19 inhibitors caused the formation of large vesicular structures inside both WT and dKO 293 cells, indicating that this Cmpd3-specific effect was unrelated to CDK8/19 activity. Hence, both Cmpd4 and Cmpd3 possess off-target activities that could potentially explain their in vivo toxicity.

### 3.4. Basal STAT1 S727 Phosphorylation Is an Imperfect Pharmacodynamic Marker of CDK8/19 Activity

Cmpd3 and Cmpd4 were used in mouse studies of Clarke et al. at doses ranging from 20 to 100 mg/kg [13], in striking contrast to 0.16 mg/kg dose used in leukemia models with cortistatin A [10], a CDK8/19 inhibitor with similar potency to Cmpd3 and Cmpd4. The dose selection of CDK8/19 inhibitors in the study of Clarke et al. was driven primarily by the effect on STAT1 phosphorylation at S727 in xenograft tumors and (in the case of rats) in spleens, which was assumed to be a pharmacodynamic (PD) marker of CDK8/19 activity. Indeed, CDK8 was demonstrated to be responsible for IFN-γ-induced STAT1 S727 phosphorylation in mouse embryo fibroblasts (MEF) [25], although low-level induction of STAT1 S727 phosphorylation by IFNγ was detectable in HCT116 cells with double knockout of CDK8 and CDK19 [26]. In contrast to IFNγ-treated cells, the knockdown of either CDK8 or Cyclin C, the binding partner of both CDK8 and CDK19, had no effect on basal STAT1 S727 phosphorylation in untreated MEF [25]. Similarly, we did not detect any effect of Senexin B on basal STAT1 S727 phosphorylation in rat neurons in culture (not shown) but basal STAT1 S727 phosphorylation in natural killer (NK) cells was shown to be inhibited by CDK8 knockdown [27]. In contrast to these varying results in normal tissues, we observed that Senexin B inhibited basal STAT1 S727 phosphorylation in all the tested tumor cell lines of different types (patent application US 2017/0115308A1), and similar results were reported in various transformed cell lines by others [11,13]. However, we also noticed that the magnitude of the inhibition of basal STAT1 S727 phosphorylation by Senexin B varied somewhat among different experiments, conducted at different times, suggesting the existence of factors other than CDK8/19 that determined STAT1 S727 phosphorylation. 

**Figure 7:**
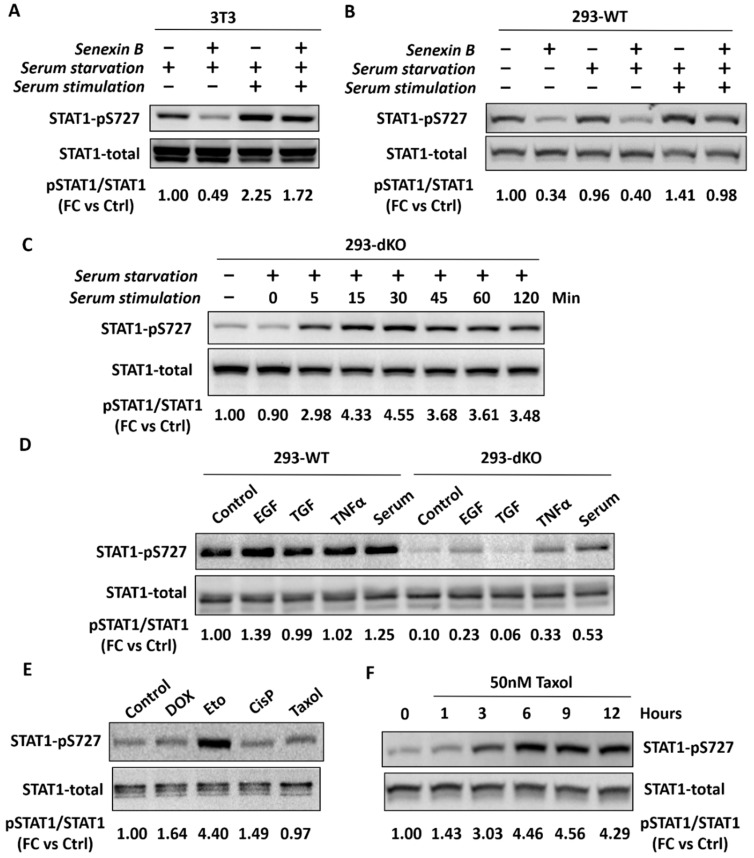
CDK8/19-dependent and independent induction of STAT1 S727 phosphorylation. Numbers below the western blot images indicate relative signal intensity of STAT1 p727 normalized to total STAT1 and presented as fold change (FC) relative to the control (Ctrl) samples. (**A**) The effect of Senexin B (1 μM) on STAT1 S727 phosphorylation in NIH 3T3 cells upon serum starvation for 24 h and after serum stimulation for 1 h (Senexin B or DMSO control were added with serum). (**B**) The effect of Senexin B (1 μM) on STAT1 S727 phosphorylation in 293-WT cells grown under regular conditions, upon serum starvation for 24 h and after serum stimulation for 1 h in 293-WT cells. (**C**) The effect of serum stimulation for the indicated periods of time on STAT1 S727 phosphorylation in 293-dKO cells. (**D**) STAT1 S727 phosphorylation in 293-WT and 293-dKO cells induced by 20 ng/mL EGF, 50 pM TGFβ, 40 ng/mL TNFα and 10% serum for 1 h, after 24 h serum starvation. (**E**) STAT1 S727 phosphorylation in 293-dKO cells induced by 3 h treatment with 2 μM doxorubicin (DOX); 50 μM etoposide (Eto); 50 μM cisplatin (CisP); 50 nM Taxol. (**F**) STAT1 S727 phosphorylation in 293-dKO cells induced by treatment with 50 nM Taxol for the indicated periods of time.

We have therefore examined different factors that could affect basal STAT1 S727 phosphorylation and took advantage of the availability of 293 dKO cells to identify signals that induce such phosphorylation in CDK8/19-independent manner. One such factor turned out to be serum stimulation. As shown in Figure 7A, Senexin B inhibits STAT1 S727 phosphorylation in serum-starved murine NIH 3T3 cells, but the addition of serum increases this phosphorylation, at which point Senexin B has only a minor effect on STAT1 S727 phosphorylation in these quasi-normal cells. Similarly, in WT 293 cells, Senexin B inhibits STAT1 S727 phosphorylation in cells growing under standard conditions and in serum-starved cells but serum stimulation increases such phosphorylation and this increase is only weakly affected by Senexin B (Figure 7B). Most importantly, serum stimulation produces time-dependent increase in STAT1 S727 phosphorylation in 293 dKO cells (Figure 7C), demonstrating that this effect of serum is independent of CDK8/19. 

The effects of different cytokines on STAT1 S727 phosphorylation in WT and dKO 293 cells are shown in Figure 7D. While the tested cytokines had little effect in the WT cells, the lower basal STAT1 S727 phosphorylation in the dKO cells (lacking CDK8/19) was increased by EGF, TNFα and serum. Figure 7E shows that CDK8/19-independent STAT1 S727 phosphorylation in dKO 293 cells was also increased by cytotoxic drugs etoposide and Taxol; the time course of the induction of CDK8/19-independent STAT1 S727 phosphorylation by 50 nM Taxol in dKO 293 cells is shown in Figure 7F.

These results indicate that IFN-γ-independent STAT1 S727 phosphorylation is induced not only by CDK8/19 but also by various cytokines and stress-inducing agents, in a CDK8/19-independent manner. It is difficult to predict how the inhibition of both on-target and off-target activities by various CDK8/19 inhibitors, especially upon prolonged in vivo treatment, would impact the variety of factors that induce STAT1 S727 phosphorylation, and therefore such phosphorylation does not provide a reliable PD marker for CDK8/19 inhibition.

## 4. Discussion

In this study, we have compared the toxicity of Cmpd3 and Cmpd4, two CDK8/19 inhibitors found to have major systemic toxicity in mammals [13], with three other CDK8/19 inhibitors for which no such toxicity was reported, Senexin B, dCA and 15w. The results of our study argue that the systemic toxicity of Cmpd3 and Cmpd4 is not due to CDK8/19 inhibition. (i) Zebrafish assays revealed a striking toxicity of Cmpd4, which was not shared by the other CDK8/19 inhibitors (although some moderate but significant toxicity was also observed in this assay at higher concentrations of Cmpd3 and dCA). This result indicates that Cmpd4 has unique activities that cause toxicity in this model and that its toxicity is not a general consequence of CDK8/19 inhibition. (ii) The toxicities of Cmpd4 and Cmpd3 relative to the other CDK8/19 inhibitors were not due to differential effects on CDK8 versus CDK19, since both compounds, as well as the other three CDK8/19 inhibitors showed similar potencies against CDK8 and CDK19 in the cell-based assay. (iii) The toxicity of Cmpd3 and Cmpd4 was not due to stronger inhibition of CDK8/19, as dCA and 15w showed similar low-nanomolar activity in the cell-based assay. (iv) We also tested if the toxicity could be due to the effects of sustained long-term inhibition of CDK8/19, but such sustained inhibition was found in wash-off assays to be associated only with dCA, which was not the most toxic compound. These findings indicate that the toxicity of Cmpd3 and Cmpd4 was not a general consequence of CDK8/19 inhibition, leaving off-target effects as the most likely cause of the systemic toxicity of Cmpd3 and Cmpd4.

Indeed, kinome profiling followed by Kd determination revealed significant off-target activities of both Cmpd3 and Cmpd4, which could potentially account for their systemic toxicity. This profiling was carried out via the DiscoverX ATP analog binding competition assay, which has been previously used to characterize the selectivity of other CDK8/19 inhibitors (Cmpd3 and Cmpd4 were shown to bind to the ATP pocket of CDK8 [13]). Kinome profiling showed that Cmpd3 had the strongest off-target effects on PIKFYVE, JNK1 and STK16 and Cmpd4 on GSK3B and GSK3A. The strongest off-target effects, those of Cmpd4 on GSK3B and of Cmpd3 on PIKFYVE, were tested and verified by independent assays. The striking induction of intracellular vesicle formation by Cmpd3 could conceivably provide a cause of toxicity, whereas inhibition of GSK3B, an off-target of Cmpd4, has been associated with significant toxicities [28,29], including the induction of bone lesions [28] that appear to be similar to those observed by Clarke et al. [13]. We note that the inhibition of these kinases is not necessarily responsible for the systemic toxicities observed in zebrafish and mammalian assays, since off-target effects can also involve kinases that are not represented in the panel, as well as non-kinase targets.

Surprisingly, both Cmpd3 and Cmpd4 showed much weaker potency in competing for ATP pocket binding of CDK8 and CDK19 in the DiscoverX assay than their CDK8/19 inhibitory activities observed in cell-based assays or in the previously reported in vitro kinase activity inhibition assays. Notably, DiscoverX, in contrast to the other assays, uses CDK8 and CDK19 without Cyclin C. Nevertheless, no discrepancy between the results of DiscoverX and other assays was seen with Senexin B or cortistatin A [8,21]. Although the Kd value for 15w in the DiscoverX assay [17] was higher than in the cell based assay, the discrepancy was not as great as in the case of Cmpd3 and Cmpd4. The unique binding mode of Cmpd3 and Cmpd4 did not translate into increased stability of CDK8/19 inhibition in cell-based assays, and it is unclear how this effect on CDK8/19 could be related to systemic toxicity. On the other hand, the special binding mode shared by chemically distinct Cmpd3 and Cmpd4 suggests possible conformational similarities, which could be related to the similar cell-based screens (based on WNT inhibition) used to isolate the first compounds in these series.

Off-target effects, even if toxic, need not necessarily be a barrier to the use of a compound, as long as its doses are judiciously selected to be restricted to on-target activity. The off-target kinase effects of Cmpd3 and Cmpd4 are weaker than their effects against CDK8/19 and, in principle, could have been minimized in vivo through the use of lower doses of the compounds. However, in vivo doses of these compounds used by Clarke et al. [13] were over two orders of magnitude higher than the therapeutic dose of cortistatin A [10], which has a similar potency against CDK8/19. The total plasma concentrations of Cmpd3 in mice treated with 30 mg/kg/d doses were 14.1 μM after 1 h, 6.9 μM after 2 h and 0.8 μM after 6 h [15], making off-target inhibition and toxicity very likely. The dose selection in the study of Clarke et al. [13] was driven by reliance on STAT1 S727 phosphorylation in tumor xenografts as a PD marker of CDK8/19 activity. While CDK8/19 is the principal determinant of IFN-γ-induced STAT1 S727 phosphorylation [25] and one of the major determinants of basal phosphorylation in the absence of IFN-γ, it is not the sole determinant of basal STAT1 S727 phosphorylation, as has been known from earlier studies [30] and demonstrated here by our analysis of 293 cells with the knockout of both CDK8 and CDK19. In particular, we have identified several cytokines and stress-inducing agents, including cytotoxic drugs, which induce STAT1 S727 phosphorylation even in the cells with the knockout of both CDK8 and CDK19. In some cases, the effect of a CDK8/19 inhibitor on STAT1 S727 phosphorylation, while apparent in control cells, becomes virtually undetectable in cells that are exposed to treatments that affect this phosphorylation. In the case of tumors subjected to prolonged treatment with CDK8/19 inhibitors, the nature of various factors that affect such tumors and that can induce CDK8/19-independent STAT1 S727 phosphorylation is unpredictable, indicating that such phosphorylation is not a reliable marker of CDK8/19 inhibition.

Given the absence of a known phosphorylation substrate that would be specific for CDK8/19, we believe that a more reliable PD approach consists of measuring the expression of genes that are regulated by CDK8/19 in a given cell type. An example of such a marker is the GREB1 gene in estrogen receptor (ER)-positive breast cancers, which is coregulated by ER and CDK8/19 in this tumor type [9]. A judicious choice of carefully validated PD markers is critical for the development of targeted inhibitors in general and CDK8/19 inhibitors in particular, allowing one to avoid inappropriate dose selection and off-target toxicity.

## Figures and Tables

**Table 1:** Kd values (nM) for the inhibition of nucleotide binding to the indicated kinases. The most strongly inhibited off-target kinases are shown in red. N.D., not done.

DiscoveRx Gene Symbol	Entrez Gene Symbol	Senexin B	CMPD3	CMPD4
CDK11	CDK19	23	59	94
CDK8	CDK8	59	190	115
MAP4K2	MAP4K2	570	N.D.	N.D.
STK16	STK16	2200	420	N.D.
PIKFYVE	PIKFYVE	14,000	100	N.D.
JNK1	MAPK8	7200	390	N.D.
GSK3A	GSK3A	>30,000	N.D.	450
GSK3B	GSK3B	N.D.	N.D.	190

## Supplementary Materials

The following are available online at https://www.mdpi.com/2073-4409/8/11/1413/s1, Figure S1: Representative images of compound-treated 24 hpf AB zebrafish embryos, Figure S2: QPCR quantitation of mRNA expression of CDK8/19-dependent genes, Figure S3: Effects of different concentrations of CDK8/19 inhibitors on ATP pocket binding of indicated kinases in KdELECT assays (Discover X), Table S1: QPCR primer sequences, Table S2: Statistics of TU zebrafish embryo bioassay, Table S3: Effects of 2 μM of Senexin B, Cmpd3 and Cmpd4 on the indicated kinases in DiscoverX assay (% control activity).
