# Supplementary material for: Systemic Toxicity Reported for CDK8/19 Inhibitors CCT251921 and MSC2530818 Is Not Due to Target Inhibition
Source: Cells. 2019 Nov 9;8(11):1413. doi: 10.3390/cells8111413 (PMC6912361; doi:10.3390/cells8111413)
Supplement: Supplementary file 1 [file cells-08-01413-s001.pdf]

Supplementary Materials

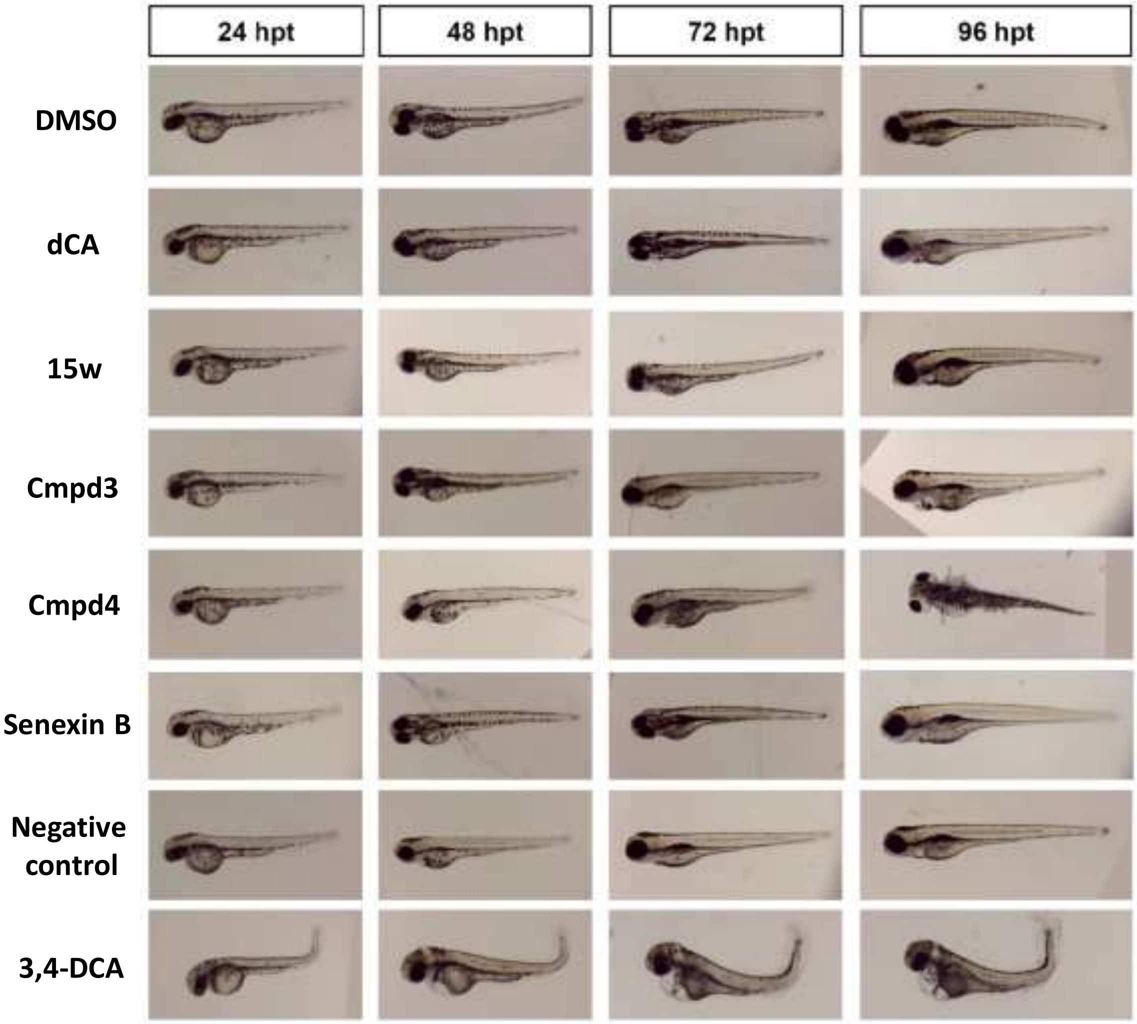

Supplemental Figure S1. Representative images of compound-treated 24 hpf AB zebrafish embryos.

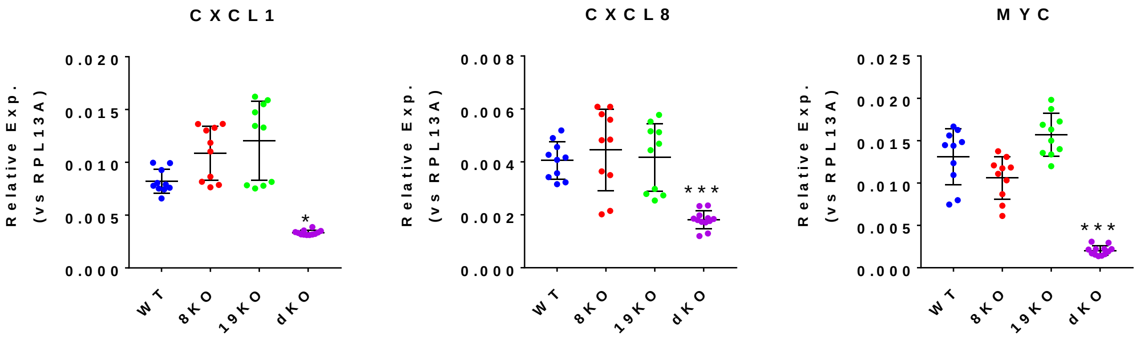

Supplemental Figure S2. QPCR quantitation of mRNA expression of CDK8/19-dependent genes. 293-WT, 8KO, 19KO and dKO cells were treated with vehicle (0.1% DMSO). Statistical significance is marked as: $p < 0.05$ (*); $p < 0.01$ (***).

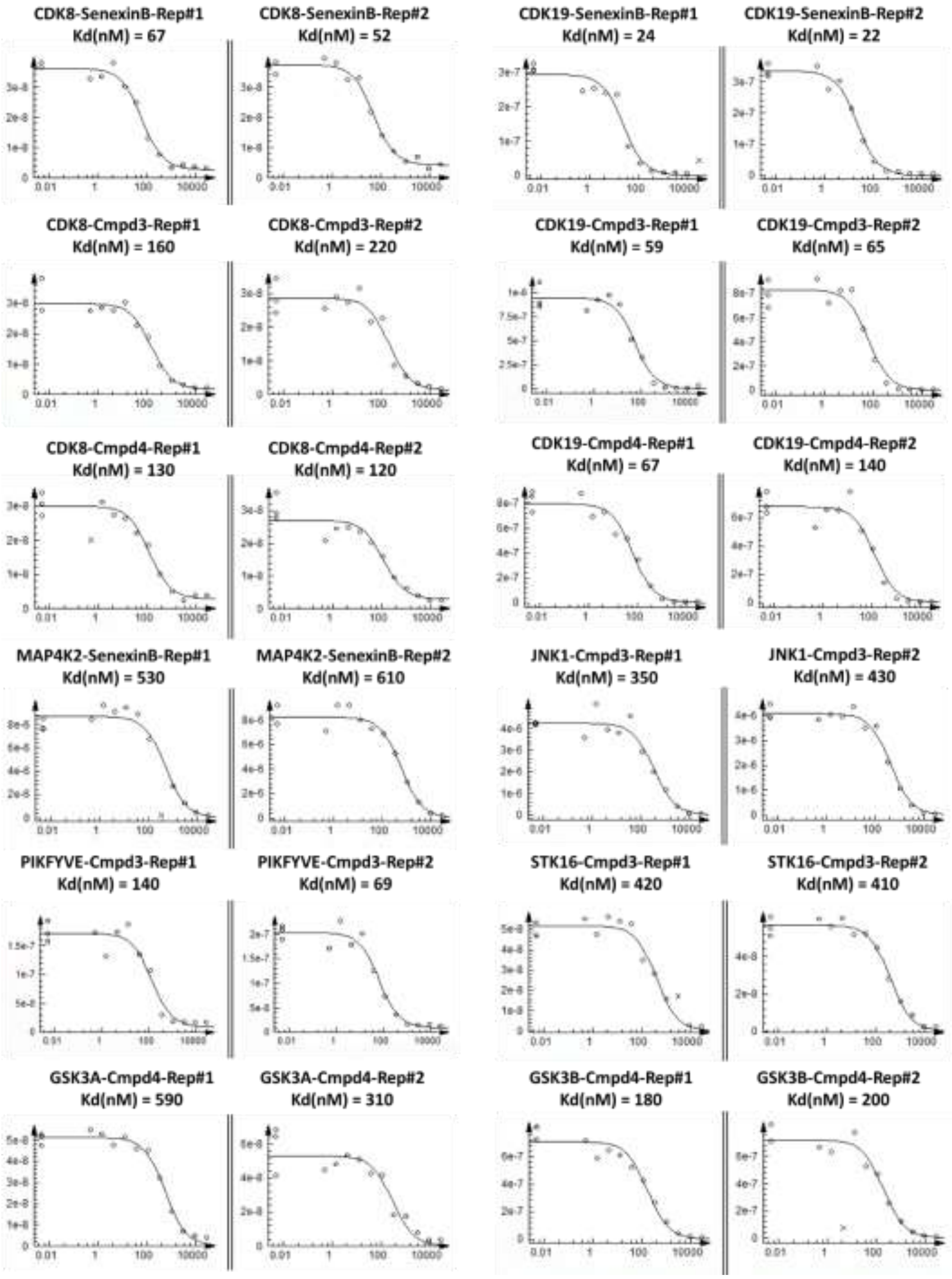

Supplemental Figure S3. Effects of different concentrations of CDK8/19 inhibitors on ATP pocket binding of indicated kinases in KdELECT assays (Discover X). Kinase ATP binding-site competition assays were conducted in duplicates. The amount of kinase target (not competed by tested compounds) measured by QPCR (Signal; y-axis) is plotted against the corresponding compound concentration in nM on log10 scale (x-axis). Data points marked with an "x" were not used for Kd determination.

Supplemental Table S1. QPCR primer sequences

**RPL13A-F**	GGCCCAGCAGTACCTGTTTA
**RPL13A-R**	AGATGGCGGAGGTGCAG
**GAPDH-F**	CCATCACCATCTTCCAGGAGCG
**GAPDH-R**	AGAGATGATGACCCTTTTGGC
**CXCL1-F**	GAAAGCTTGCCTCAATCCTG
**CXCL1-R**	AACAGCCACCAGTGAGCTTC
**CXCL8-F**	TCCTGATTTCTGCAGCTCTGT
**CXCL8-R**	AAATTTGGGGTGGAAAGGTT
**MYC-F**	CCAACAGGAACTATGACCTCGACTAC
**MYC-R**	CTCGAATTTCTTCCAGATATCCT
**JUN-F**	GGAGACAAGTGGCAGAGTCC
**JUN-R**	GTCCTTCTTCTCTTGCGTGG
**ADAMTS1-F**	GAGGACGAAGGGACTGAGG
**ADAMTS1-R**	AGCGGTGACTGGACACAAAT

16

Supplemental Table S2. Statistics of TU zebrafish embryo bioassay

	Day 3				Day 4				Day 5			
Drug	p.value	Relative Risk (RR) estimate	95% CI.lower	95% CI.upper	p.value	Relative Risk (RR) estimate	95% CI.lower	95% CI.upper	p.value	Relative Risk (RR) estimate	95% CI.lower	95% CI.upper
2uM Senexin B	0.483	0.00	0.00	NA	0.876	1.18	0.15	9.17	0.236	2.06	0.63	6.79
5uM Senexin B	0.016	3.94	1.18	13.11	0.023	2.89	1.11	7.52	0.016	2.44	1.16	5.13
1uM Cmpd#3	0.343	0.00	0.00	NA	0.007	3.86	1.36	10.92	0.008	3.00	1.30	6.90
2uM Cmpd#3	0.000	9.71	3.41	27.64	0.000	6.10	2.70	13.80	0.000	4.37	2.31	8.26
0.5uM Cmpd#4	0.000	12.20	4.25	35.00	0.000	13.94	6.48	29.98	0.000	13.15	7.50	23.05
1uM Cmpd#4	0.000	37.13	13.87	99.41	0.000	27.64	13.33	57.30	0.000	16.13	9.30	27.96
2uM Cmpd#4	0.000	46.47	17.57	122.91	0.000	28.29	13.66	58.55	0.000	16.50	9.54	28.55
1uM CsA	0.080	3.45	0.80	14.87	0.001	4.60	1.70	12.45	0.007	3.07	1.34	7.05
2uM CsA	0.000	8.61	2.87	25.81	0.000	7.38	3.22	16.90	0.000	4.54	2.33	8.87
1uM 15w	0.544	0.00	0.00	NA	0.665	1.57	0.20	12.07	0.931	0.92	0.13	6.65
2uM 15w	0.000	8.74	2.60	29.34	0.001	4.99	1.79	13.95	0.001	3.88	1.71	8.79

17

Supplemental Table S3. Effects of 2 µM of Senexin B, Cmpd3 and Cmpd4 on the indicated kinases in DiscoverX assay (% control activity)

DiscoveRx Gene Symbol	Entrez Gene Symbol	2µM Senexin B	2µM Cmpd3	2µM Cmpd4
AAK1	AAK1	100	87	100
ABL1(E255K)-phosphorylated	ABL1	100	62	99
ABL1(F317I)-nonphosphorylated	ABL1	100	71	91
ABL1(F317I)-phosphorylated	ABL1	68	67	75
ABL1(F317L)-nonphosphorylated	ABL1	98	82	100
ABL1(F317L)-phosphorylated	ABL1	90	71	63
ABL1(H396P)-nonphosphorylated	ABL1	78	98	100
ABL1(H396P)-phosphorylated	ABL1	97	53	94
ABL1(M351T)-phosphorylated	ABL1	81	83	71
ABL1(Q252H)-nonphosphorylated	ABL1	82	98	100
ABL1(Q252H)-phosphorylated	ABL1	100	58	72
ABL1(T315I)-nonphosphorylated	ABL1	87	85	100
ABL1(T315I)-phosphorylated	ABL1	100	68	65
ABL1(Y253F)-phosphorylated	ABL1	100	72	87
ABL1-nonphosphorylated	ABL1	89	96	94
ABL1-phosphorylated	ABL1	100	84	100
ABL2	ABL2	94	99	100
ACVR1	ACVR1	96	100	100
ACVR1B	ACVR1B	80	15	98
ACVR2A	ACVR2A	71	100	47
ACVR2B	ACVR2B	90	94	100
ACVRL1	ACVRL1	100	100	93
ADCK3	CABC1	100	77	100
ADCK4	ADCK4	65	100	94
AKT1	AKT1	100	96	95

AKT2	AKT2	100	89	90
AKT3	AKT3	93	100	100
ALK	ALK	100	32	97
ALK(C1156Y)	ALK	NA	58	100
ALK(L1196M)	ALK	NA	92	94
AMPK-alpha1	PRKAA1	100	100	93
AMPK-alpha2	PRKAA2	100	89	81
ANKK1	ANKK1	87	78	86
ARK5	NUAK1	100	90	80
ASK1	MAP3K5	100	100	99
ASK2	MAP3K6	89	75	71
AURKA	AURKA	100	78	83
AURKB	AURKB	82	72	72
AURKC	AURKC	80	100	95
AXL	AXL	99	75	96
BIKE	BMP2K	92	74	98
BLK	BLK	100	100	100
BMPR1A	BMPR1A	72	13	100
BMPR1B	BMPR1B	84	85	82
BMPR2	BMPR2	93	69	78
BMX	BMX	100	68	94
BRAF	BRAF	100	73	82
BRAF(V600E)	BRAF	100	63	89
BRK	PTK6	100	100	100
BRSK1	BRSK1	87	100	98
BRSK2	BRSK2	100	91	100
BTK	BTK	100	68	93
BUB1	BUB1	100	46	76
CAMK1	CAMK1	96	72	81

CAMK1B	PNCK	NA	92	98
CAMK1D	CAMK1D	100	97	96
CAMK1G	CAMK1G	100	73	96
CAMK2A	CAMK2A	80	93	92
CAMK2B	CAMK2B	100	87	91
CAMK2D	CAMK2D	100	81	73
CAMK2G	CAMK2G	100	81	100
CAMK4	CAMK4	100	100	100
CAMKK1	CAMKK1	100	86	97
CAMKK2	CAMKK2	100	82	100
CASK	CASK	99	73	71
CDC2L1	CDK11B	100	98	100
CDC2L2	CDC2L2	100	97	100
CDC2L5	CDK13	100	64	56
CDK11	CDK19	1.4	1.8	4.4
CDK2	CDK2	92	92	96
CDK3	CDK3	100	96	100
CDK4	CDK4	NA	67	57
CDK4-cyclinD1	CDK4	100	90	92
CDK4-cyclinD3	CDK4	100	66	79
CDK5	CDK5	100	100	98
CDK7	CDK7	92	84	92
CDK8	CDK8	2.2	23	33
CDK9	CDK9	100	99	100
CDKL1	CDKL1	100	82	86
CDKL2	CDKL2	50	98	100
CDKL3	CDKL3	77	95	100
CDKL5	CDKL5	100	70	94
CHEK1	CHEK1	100	88	100

CHEK2	CHEK2	100	90	95
CIT	CIT	70	84	100
CLK1	CLK1	65	93	65
CLK2	CLK2	80	88	33
CLK3	CLK3	92	65	62
CLK4	CLK4	41	89	69
CSF1R	CSF1R	94	63	100
CSF1R-autoinhibited	CSF1R	84	34	69
CSK	CSK	100	59	86
CSNK1A1	CSNK1A1	73	100	100
CSNK1A1L	CSNK1A1L	90	85	96
CSNK1D	CSNK1D	67	83	85
CSNK1E	CSNK1E	95	87	78
CSNK1G1	CSNK1G1	70	98	91
CSNK1G2	CSNK1G2	75	98	98
CSNK1G3	CSNK1G3	81	94	98
CSNK2A1	CSNK2A1	100	91	69
CSNK2A2	CSNK2A2	100	89	96
CTK	MATK	98	100	100
DAPK1	DAPK1	86	98	100
DAPK2	DAPK2	87	93	93
DAPK3	DAPK3	68	95	100
DCAMKL1	DCLK1	90	43	70
DCAMKL2	DCLK2	100	100	100
DCAMKL3	DCLK3	86	100	47
DDR1	DDR1	100	78	100
DDR2	DDR2	94	78	79
DLK	MAP3K12	100	61	81
DMPK	DMPK	99	94	98

DMPK2	CDC42BPG	100	100	93
DRAK1	STK17A	86	100	100
DRAK2	STK17B	100	96	100
DYRK1A	DYRK1A	100	63	81
DYRK1B	DYRK1B	67	88	79
DYRK2	DYRK2	77	80	90
EGFR	EGFR	100	89	100
EGFR(E746-A750del)	EGFR	100	80	84
EGFR(G719C)	EGFR	100	76	68
EGFR(G719S)	EGFR	100	82	66
EGFR(L747-E749del, A750P)	EGFR	99	85	100
EGFR(L747-S752del, P753S)	EGFR	100	95	92
EGFR(L747-T751del,Sins)	EGFR	81	72	71
EGFR(L858R)	EGFR	100	79	100
EGFR(L858R,T790M)	EGFR	100	81	51
EGFR(L861Q)	EGFR	78	78	73
EGFR(S752-I759del)	EGFR	72	77	51
EGFR(T790M)	EGFR	91	82	70
EIF2AK1	EIF2AK1	100	73	90
EPHA1	EPHA1	93	93	100
EPHA2	EPHA2	83	80	89
EPHA3	EPHA3	80	98	100
EPHA4	EPHA4	99	99	90
EPHA5	EPHA5	100	98	100
EPHA6	EPHA6	97	100	97
EPHA7	EPHA7	100	95	100
EPHA8	EPHA8	100	98	100
EPHB1	EPHB1	91	100	93
EPHB2	EPHB2	89	94	88

EPHB3	EPHB3	100	100	100
EPHB4	EPHB4	96	91	97
EPHB6	EPHB6	84	84	87
ERBB2	ERBB2	83	70	64
ERBB3	ERBB3	87	95	90
ERBB4	ERBB4	98	99	100
ERK1	MAPK3	94	100	100
ERK2	MAPK1	100	97	100
ERK3	MAPK6	100	100	100
ERK4	MAPK4	100	100	100
ERK5	MAPK7	100	100	100
ERK8	MAPK15	100	96	45
ERN1	ERN1	100	32	59
FAK	PTK2	93	100	97
FER	FER	100	100	100
FES	FES	100	96	100
FGFR1	FGFR1	88	100	98
FGFR2	FGFR2	100	91	97
FGFR3	FGFR3	100	72	99
FGFR3(G697C)	FGFR3	73	95	100
FGFR4	FGFR4	100	100	100
FGR	FGR	95	91	87
FLT1	FLT1	80	100	98
FLT3	FLT3	100	75	100
FLT3(D835H)	FLT3	100	83	100
FLT3(D835V)	FLT3	NA	37	50
FLT3(D835Y)	FLT3	85	98	100
FLT3(ITD)	FLT3	54	100	97
FLT3(ITD,D835V)	FLT3	NA	59	70

FLT3(ITD,F691L)	FLT3	NA	40	54
FLT3(K663Q)	FLT3	100	78	100
FLT3(N841I)	FLT3	63	88	100
FLT3(R834Q)	FLT3	100	59	66
FLT3-autoinhibited	FLT3	100	69	75
FLT4	FLT4	100	98	100
FRK	FRK	100	100	100
FYN	FYN	100	100	100
GAK	GAK	85	74	100
GCN2(Kin.Dom.2,S808G)	EIF2AK4	100	100	62
GRK1	GRK1	64	82	90
GRK2	ADRBK1	NA	91	99
GRK3	ADRBK2	NA	96	100
GRK4	GRK4	100	52	94
GRK7	GRK7	80	87	96
GSK3A	GSK3A	97	97	17
GSK3B	GSK3B	85	69	43
HASPIN	GSG2	36	66	70
HCK	HCK	85	100	100
HIPK1	HIPK1	75	94	100
HIPK2	HIPK2	72	72	75
HIPK3	HIPK3	55	84	84
HIPK4	HIPK4	73	100	99
HPK1	MAP4K1	100	93	86
HUNK	HUNK	72	100	100
ICK	ICK	93	73	67
IGF1R	IGF1R	100	96	100
IKK-alpha	CHUK	100	76	73
IKK-beta	IKBKB	100	66	70

IKK-epsilon	IKBKE	80	81	90
INSR	INSR	98	83	81
INSRR	INSRR	100	91	99
IRAK1	IRAK1	67	67	89
IRAK3	IRAK3	83	90	86
IRAK4	IRAK4	100	75	79
ITK	ITK	100	100	100
JAK1(JH1domain-catalytic)	JAK1	100	90	48
JAK1(JH2domain-pseudokinase)	JAK1	89	42	80
JAK2(JH1domain-catalytic)	JAK2	85	68	49
JAK3(JH1domain-catalytic)	JAK3	71	63	55
JNK1	MAPK8	99	23	70
JNK2	MAPK9	100	66	83
JNK3	MAPK10	100	48	68
KIT	KIT	82	100	100
KIT(A829P)	KIT	73	79	91
KIT(D816H)	KIT	79	56	47
KIT(D816V)	KIT	100	88	95
KIT(L576P)	KIT	86	99	99
KIT(V559D)	KIT	70	97	100
KIT(V559D,T670I)	KIT	79	76	100
KIT(V559D,V654A)	KIT	100	100	100
KIT-autoinhibited	KIT	100	75	79
LATS1	LATS1	100	100	53
LATS2	LATS2	78	70	61
LCK	LCK	90	100	100
LIMK1	LIMK1	99	100	98
LIMK2	LIMK2	100	100	89
LKB1	STK11	100	100	100

LOK	STK10	100	100	100
LRRK2	LRRK2	99	59	67
LRRK2(G2019S)	LRRK2	70	58	63
LTK	LTK	92	62	99
LYN	LYN	80	97	85
LZK	MAP3K13	100	93	91
MAK	MAK	99	100	100
MAP3K1	MAP3K1	94	74	75
MAP3K15	MAP3K15	90	99	51
MAP3K2	MAP3K2	100	68	74
MAP3K3	MAP3K3	97	97	95
MAP3K4	MAP3K4	74	100	100
MAP4K2	MAP4K2	31	64	74
MAP4K3	MAP4K3	97	100	100
MAP4K4	MAP4K4	91	100	70
MAP4K5	MAP4K5	97	100	51
MAPKAPK2	MAPKAPK2	100	86	100
MAPKAPK5	MAPKAPK5	100	67	72
MARK1	MARK1	82	99	100
MARK2	MARK2	82	97	100
MARK3	MARK3	90	100	100
MARK4	MARK4	100	100	100
MAST1	MAST1	100	97	98
MEK1	MAP2K1	92	79	72
MEK2	MAP2K2	96	78	76
MEK3	MAP2K3	74	57	69
MEK4	MAP2K4	100	54	86
MEK5	MAP2K5	100	81	70
MEK6	MAP2K6	100	83	93

MELK	MELK	94	86	100
MERTK	MERTK	100	88	91
MET	MET	92	92	99
MET(M1250T)	MET	100	99	100
MET(Y1235D)	MET	93	72	68
MINK	MINK1	96	75	83
MKK7	MAP2K7	100	78	67
MKNK1	MKNK1	66	67	70
MKNK2	MKNK2	95	76	79
MLCK	MYLK3	89	100	98
MLK1	MAP3K9	100	95	100
MLK2	MAP3K10	92	99	89
MLK3	MAP3K11	96	97	95
MRCKA	CDC42BPA	90	100	99
MRCKB	CDC42BPB	96	90	100
MST1	STK4	100	100	100
MST1R	MST1R	100	99	91
MST2	STK3	83	99	96
MST3	STK24	100	93	100
MST4	MST4	100	84	68
MTOR	MTOR	76	87	94
MUSK	MUSK	100	55	100
MYLK	MYLK	83	94	100
MYLK2	MYLK2	99	87	92
MYLK4	MYLK4	79	79	94
MYO3A	MYO3A	100	100	100
MYO3B	MYO3B	100	100	100
NDR1	STK38	100	73	78
NDR2	STK38L	100	100	100

NEK1	NEK1	97	71	73
NEK10	NEK10	NA	53	63
NEK11	NEK11	88	68	62
NEK2	NEK2	92	97	96
NEK3	NEK3	96	79	81
NEK4	NEK4	91	80	78
NEK5	NEK5	100	100	100
NEK6	NEK6	93	97	95
NEK7	NEK7	100	100	93
NEK9	NEK9	100	98	100
NIK	MAP3K14	NA	64	42
NIM1	MGC42105	100	70	75
NLK	NLK	100	96	100
OSR1	OXSR1	100	77	99
p38-alpha	MAPK14	100	95	97
p38-beta	MAPK11	77	71	95
p38-delta	MAPK13	100	88	100
p38-gamma	MAPK12	100	85	99
PAK1	PAK1	100	80	51
PAK2	PAK2	100	79	68
PAK3	PAK3	100	100	100
PAK4	PAK4	100	95	100
PAK6	PAK6	100	56	100
PAK7	PAK7	100	100	100
PCTK1	CDK16	100	73	78
PCTK2	CDK17	100	100	100
PCTK3	CDK18	100	95	100
PDGFRA	PDGFRA	100	67	75
PDGFRB	PDGFRB	94	100	100

PDPK1	PDPK1	100	100	92
PFCDPK1(P.falciparum)	CDPK1	100	68	68
PFPK5(P.falciparum)	MAL13P1.279	100	100	100
PFTAIRE2	CDK15	100	100	100
PFTK1	CDK14	100	100	99
PHKG1	PHKG1	95	100	93
PHKG2	PHKG2	100	100	100
PIK3C2B	PIK3C2B	92	57	86
PIK3C2G	PIK3C2G	100	31	81
PIK3CA	PIK3CA	93	82	94
PIK3CA(C420R)	PIK3CA	62	87	85
PIK3CA(E542K)	PIK3CA	77	85	58
PIK3CA(E545A)	PIK3CA	90	90	86
PIK3CA(E545K)	PIK3CA	61	89	67
PIK3CA(H1047L)	PIK3CA	94	90	95
PIK3CA(H1047Y)	PIK3CA	72	100	84
PIK3CA(I800L)	PIK3CA	95	77	46
PIK3CA(M1043I)	PIK3CA	80	71	76
PIK3CA(Q546K)	PIK3CA	100	78	78
PIK3CB	PIK3CB	89	100	100
PIK3CD	PIK3CD	73	65	88
PIK3CG	PIK3CG	59	65	94
PIK4CB	PI4KB	100	23	77
PIKFYVE	PIKFYVE	NA	0	80
PIM1	PIM1	100	86	100
PIM2	PIM2	100	100	100
PIM3	PIM3	100	87	75
PIP5K1A	PIP5K1A	100	87	75
PIP5K1C	PIP5K1C	81	77	95

PIP5K2B	PIP4K2B	97	89	60
PIP5K2C	PIP4K2C	54	86	100
PKAC-alpha	PRKACA	100	100	91
PKAC-beta	PRKACB	86	100	91
PKMYT1	PKMYT1	84	92	100
PKN1	PKN1	100	100	94
PKN2	PKN2	78	98	99
PKNB(M.tuberculosis)	pknB	99	85	72
PLK1	PLK1	93	72	70
PLK2	PLK2	100	85	85
PLK3	PLK3	100	81	75
PLK4	PLK4	100	92	89
PRKCD	PRKCD	92	96	97
PRKCE	PRKCE	65	43	61
PRKCH	PRKCH	100	92	85
PRKCI	PRKCI	100	87	65
PRKCQ	PRKCQ	100	85	83
PRKD1	PRKD1	58	100	88
PRKD2	PRKD2	75	96	94
PRKD3	PRKD3	100	92	100
PRKG1	PRKG1	77	100	100
PRKG2	PRKG2	100	80	86
PRKR	EIF2AK2	88	99	67
PRKX	PRKX	100	92	73
PRP4	PRPF4B	96	100	100
PYK2	PTK2B	100	84	96
QSK	KIAA0999	100	73	61
RAF1	RAF1	93	99	100
RET	RET	100	13	100

RET(M918T)	RET	100	100	100
RET(V804L)	RET	94	99	95
RET(V804M)	RET	100	100	100
RIOK1	RIOK1	95	100	100
RIOK2	RIOK2	84	75	77
RIOK3	RIOK3	100	97	100
RIPK1	RIPK1	100	100	100
RIPK2	RIPK2	84	100	98
RIPK4	RIPK4	100	69	83
RIPK5	DSTYK	100	42	79
ROCK1	ROCK1	100	82	62
ROCK2	ROCK2	100	71	73
ROS1	ROS1	98	93	100
RPS6KA4(Kin.Dom.1-N-terminal)	RPS6KA4	99	100	98
RPS6KA4(Kin.Dom.2-C-terminal)	RPS6KA4	100	79	79
RPS6KA5(Kin.Dom.1-N-terminal)	RPS6KA5	100	89	93
RPS6KA5(Kin.Dom.2-C-terminal)	RPS6KA5	100	100	100
RSK1(Kin.Dom.1-N-terminal)	RPS6KA1	99	80	100
RSK1(Kin.Dom.2-C-terminal)	RPS6KA1	100	98	100
RSK2(Kin.Dom.1-N-terminal)	RPS6KA3	100	67	77
RSK2(Kin.Dom.2-C-terminal)	RPS6KA3	100	78	92
RSK3(Kin.Dom.1-N-terminal)	RPS6KA2	100	86	93
RSK3(Kin.Dom.2-C-terminal)	RPS6KA2	96	92	96
RSK4(Kin.Dom.1-N-terminal)	RPS6KA6	100	74	81
RSK4(Kin.Dom.2-C-terminal)	RPS6KA6	100	85	100
S6K1	RPS6KB1	100	69	67
SBK1	SBK1	100	74	90
SGK	SGK1	83	59	62
SgK110	SgK110	100	100	100

SGK2	SGK2	NA	71	67
SGK3	SGK3	81	60	79
SIK	SIK1	100	96	98
SIK2	SIK2	74	100	100
SLK	SLK	100	98	98
SNARK	NUAK2	100	61	82
SNRK	SNRK	100	85	90
SRC	SRC	100	100	99
SRMS	SRMS	100	78	77
SRPK1	SRPK1	100	79	100
SRPK2	SRPK2	100	98	100
SRPK3	SRPK3	100	42	85
STK16	STK16	60	14	94
STK33	STK33	100	100	100
STK35	STK35	100	100	100
STK36	STK36	95	95	85
STK39	STK39	100	78	66
SYK	SYK	81	100	100
TAK1	MAP3K7	72	74	88
TAOK1	TAOK1	96	69	65
TAOK2	TAOK2	89	73	80
TAOK3	TAOK3	100	68	75
TBK1	TBK1	100	85	93
TEC	TEC	100	94	100
TESK1	TESK1	83	100	85
TGFBR1	TGFBR1	85	91	100
TGFBR2	TGFBR2	100	100	100
TIE1	TIE1	100	88	100
TIE2	TEK	84	63	73

TLK1	TLK1	100	94	100
TLK2	TLK2	100	98	66
TNIK	TNIK	100	95	100
TNK1	TNK1	100	87	100
TNK2	TNK2	100	84	100
TNNI3K	TNNI3K	100	100	100
TRKA	NTRK1	94	66	76
TRKB	NTRK2	100	70	84
TRKC	NTRK3	99	73	86
TRPM6	TRPM6	66	72	78
TSSK1B	TSSK1B	92	83	100
TSSK3	TSSK3	NA	100	100
TTK	TTK	73	44	91
TXK	TXK	98	96	85
TYK2(JH1domain-catalytic)	TYK2	73	59	58
TYK2(JH2domain-pseudokinase)	TYK2	90	66	65
TYRO3	TYRO3	100	100	93
ULK1	ULK1	79	71	83
ULK2	ULK2	92	72	71
ULK3	ULK3	84	65	76
VEGFR2	KDR	100	69	80
VPS34	PIK3C3	NA	56	68
VRK2	VRK2	100	61	47
WEE1	WEE1	80	98	97
WEE2	WEE2	100	84	98
WNK1	WNK1	100	78	74
WNK2	WNK2	NA	77	59
WNK3	WNK3	93	73	73
WNK4	WNK4	NA	70	71
YANK1	STK32A	100	74	87
YANK2	STK32B	98	98	78
YANK3	STK32C	100	100	91
YES	YES1	100	94	100
YSK1	STK25	86	51	91
YSK4	MAP3K19	41	63	70
ZAK	ZAK	70	99	82
ZAP70	ZAP70	79	65	55
